# An Account of the Practice of One of the Physicians of the Westminster General Dispensary, and of the Western Dispensary; from February 20 to March 20, 1806

**Published:** 1806-04

**Authors:** Samuel Fothergill

**Affiliations:** Southampton Street, Strand


### *An Account of the Practice of one of the Physicians of the Westminster General Dispensary, and of the Western Dispensary; from February 20 to March 20, 1806.*

| | | | |
|---|---|---|---|
| ACUTE DISEASES. | | Gastrodynia | 3 |
| Continued Fever | 5 | Scirrhus Liver | 1 |
| Catarrhal Fever | 3 | Jaundice | 2 |
| Epistaxis | 1 | Vomiting | 3 |
| Peripneumony | 2 | Hæmorrhoids | 1 |
| Pleurisy | 2 | Hæmatemesis | 1 |
| Acute Rheumatism | 1 | Asthenia | 6 |
| Ophthalmia | 1 | Dyspepsia | 3 |
| Hepatitis | 1 | Pyrosis | 1 |
| | | Diarrhœa | 2 |
| CHRONIC DISEASES. | | Dysuria | 2 |
| Pulmonary Consumption | 3 | Cephalalgia et Vertigo | 3 |
| Scrophula | 3 | Epilepsy | 1 |
| Hectica Senilis | 1 | Paralysis | 1 |
| Cough and Dyspnœa | 18 | Ascites | 2 |
| Pleurodynia | 2 | Anasarca | 3 |

Inflammation

| | | | |
|---|---|---|---|
| Inflammation of the Leg | 2 | Menorrhagia | 2 |
| Lumbago | 2 | Dysmenorrhœa | 1 |
| Hernia | 1 | Leucorrhœa | 4 |
| CUTANEOUS DISEASES. | | Abortion | 1 |
| Herpes | 1 | Hooping Cough | 1 |
| Prurigo | 1 | Measles | 1 |
| Psora | 2 | Fever | 1 |
| Syphilitic Eruption | 1 | Worms | 2 |
| DISEASES OF WOMEN AND CHILDREN. | | Hydrocephalus | 1 |
| Amenorrhœa | 3 | | |

I have been induced to write this account of diseases at the request of a truly respectable and eminent physician, who has stated, that "in order to have a more complete and accurate account of the prevailing diseases, it would be necessary that the practitioners who superintend the numerous medical institutions in this metropolis, should publish monthly, or quarterly, the result of their experience, since their observations must be made among the class of people most exposed to the vicissitudes of the season, and to other causes of disease."

With this view I propose to give a monthly statement of the diseases which fall under my notice, and offer such practical observations as may occur, together with any information furnished by my medical friends.

The winter has been particularly mild, and as far as my own experience goes, favourable to health, notwithstanding a remarkable degree of humidity in the atmosphere.

The month of February was unusually warm; the prevailing wind, south-east. During the greatest part of the present month, the wind has been north-east, and for some days the weather was extremely cold, with a heavy fall of snow, and much rain.

The acute diseases, usually attendant on this season, have assumed a mild character, and have generally had a favourable termination. The hooping cough has been prevalent in some families and schools, at the west end of the town; and a few cases of measles and scarlet fever have occurred. The symptoms of continued fever classed among the acute diseases of this Report, were very slight, and did not seem to originate from contagion. The cases were bu[illegible] of short duration, and all terminated favourably.

The case of hæmatemesis occurred in a woman abou[illegible] forty years of age, of a costive habit. Whilst in a stat[illegible] of debility and heat from cooking, she was exposed to th[illegible] influenc[illegible]

influence of cold, and soon after complained of pain, and a sense of weight in the stomach, with general uneasiness; this was succeeded by vomiting of blood in considerable quantity, and continued at intervals till she received relief from medicine. The abdomen was hard and rather full; she had little appetite, and looked and felt very faint. The method of treatment recommended in Dr. Hamilton's recent publication on this subject seemed suitable for the present case. She accordingly took a brisk purgative every other morning, and in the intervening day small doses of vitriolated magnesia in infusion of gentian. The vomiting ceased entirely after the second cathartic, and her recovery was soon effected.

The symptoms of hydrocephalus, on its first appearance, and those of worms in the intestinal canal, are sometimes so similar as to render it difficult to distinguish the two affections from each other; in this early stage, however, it is highly gratifying and useful to know that the remedy which will often remove the latter complaint, is also beneficial, and sometimes even effectual in the former. A child five years of age, from being active and hearty, began to droop, to toss her head about, and let her chin drop on the breast; her appetite failed; she complained of pain in the fore-part of the head and in the extremities; was sometimes fretful; at other times delirious, and had disturbed sleep, often interrupted by starting and crying out. When I saw her, these symptoms had been observed about three weeks. The pupils of the eyes were much dilated, and she seemed to take little notice of surrounding objects.

The medicine prescribed was Pulv. e. scammon. cum calomel, in doses of twelve grains, every other morning.

After the three first doses, a number of ascarides was passed, with a copious and offensive discharge, and before the end of a fortnight she was restored to perfect health.

SAMUEL FOTHERGILL.

*Southampton Street, Strand, March* 21, 1806.

